# Quantitative imaging of heterogeneous dynamics in drying and aging paints

**DOI:** 10.1038/srep34383

**Published:** 2016-09-29

**Authors:** Hanne M. van der Kooij, Remco Fokkink, Jasper van der Gucht, Joris Sprakel

**Affiliations:** 1 Physical Chemistry and Soft Matter, Wageningen University & Research, Stippeneng 4, 6708 WE Wageningen, The Netherlands; 2 Dutch Polymer Institute (DPI), P.O. Box 902, 5600 AX Eindhoven, The Netherlands

## Abstract

Drying and aging paint dispersions display a wealth of complex phenomena that make their study fascinating yet challenging. To meet the growing demand for sustainable, high-quality paints, it is essential to unravel the microscopic mechanisms underlying these phenomena. Visualising the governing dynamics is, however, intrinsically difficult because the dynamics are typically heterogeneous and span a wide range of time scales. Moreover, the high turbidity of paints precludes conventional imaging techniques from reaching deep inside the paint. To address these challenges, we apply a scattering technique, Laser Speckle Imaging, as a versatile and quantitative tool to elucidate the internal dynamics, with microscopic resolution and spanning seven decades of time. We present a toolbox of data analysis and image processing methods that allows a tailored investigation of virtually any turbid dispersion, regardless of the geometry and substrate. Using these tools we watch a variety of paints dry and age with unprecedented detail.

During the drying of particulate dispersions, such as paints or inks, a rich diversity of thermodynamic, hydrodynamic and elastic stresses emerge which govern the fate of the system^1^,^2^. As stress heterogeneities develop in both space and time, a wide range of instabilities can occur, including fracture^3^,^4^,^5^, wrinkling^6^, and the formation of pinholes^7^. Even after full evaporation of the dispersing medium, paint films continue to evolve over time, for example due to chemical curing reactions, the reorganisation of particles within the paint film^8^ and the delamination of entire paint fragments from their substrate^9^. In some cases, instabilities occur almost simultaneously with the removal of solvent from the film, while in other cases they may take hours, days or even many years to become apparent; most notably, the continuous aging of curing resins in artist oil paintings can lead to the development of surface defects centuries after the paint was applied^10^. Clearly, understanding how the fluid and particle dynamics at the microscale govern the stability, aesthetics and longevity of a painted surface is difficult as it involves a wide range of time and length scales. This is not only an important challenge for the preservation of painted surfaces and artworks but also in the development of new sustainable coatings. With the increasing demand to eradicate volatile organic compounds (VOCs) from paints, due to their detrimental effects on the environment and the health of professional painters, developing water-based, solvent-free, alternatives has become urgent. Yet, the aforementioned instabilities are particularly severe for water-based paints, because all of the functional film-forming components are present as dispersed particles in water^11^. Arriving at a deeper understanding of the relationship between microscopic dynamics and the formation and aging of a paint film is a crucial step in the endeavour towards sustainable coatings^12^. Moreover, establishing the generic origins of how drying dispersions become unstable is of fundamental importance in a much larger class of phenomena, ranging from the cracking of drying soils^13^ to the inhomogeneous deposition of solutes from droplets^14^, e.g. in inkjet printing^15^ or blood splatter^16^. Connecting the wide range of time and length scales involved in this complex problem requires methods in which the rich spatiotemporal heterogeneities can be directly and quantitatively visualised. Conventional optical microscopy is rarely suited to this task as virtually all paint films are inherently turbid, leading to multiple scattering of light and low light transmission. By contrast, while turbidity is not an issue for resonant imaging methods such as MRI, these do not offer the spatiotemporal resolution to resolve the origin of such instabilities.

In this paper, we adapt the medical imaging technique Laser Speckle Imaging (LSI) to reveal and quantify the hidden dynamics deep within drying paint films and droplets. This enables us to illuminate a complex array of dynamical processes which previously remained obscured, even for strongly scattering, light absorbing paints applied onto inhomogeneous and porous substrates such as paper or wood. In all these cases, we can extract quantitative information about flow velocities, diffusion rates and spatial correlations in heterogeneous dynamics with high spatial (micrometre) and temporal (millisecond) resolution.

## Results

### Laser Speckle Imaging

**Figure 1:**
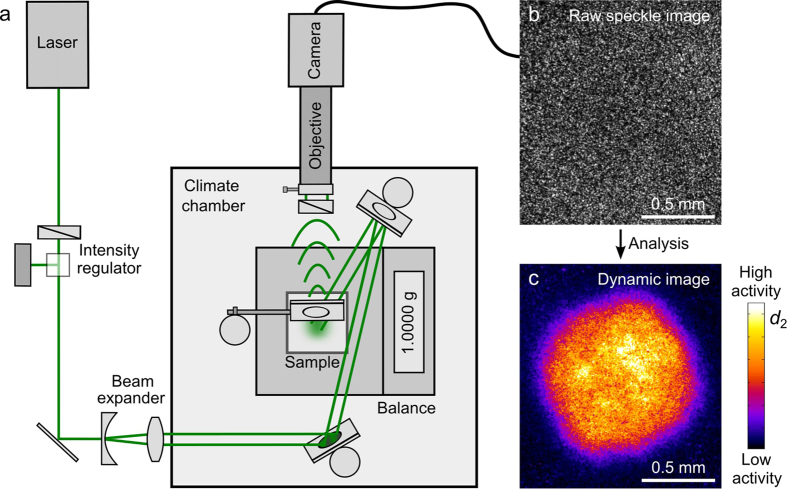
Schematic top view of the Laser Speckle Imaging set-up and output. (**a**) The sample is illuminated with an expanded laser beam, and the backscattered light is detected with a camera. This gives a speckled image of the sample (**b**), here a drying white paint droplet on white paper. The contrast from the absolute intensity is too low to reveal the location of the droplet. Instead, LSI relies on fluctuations of the speckle intensity for its contrast, caused by motion of scattering objects (see Supplementary Movie S1 for the speckle movie). These fluctuations are pixelwise translated into dynamic activities via the autocorrelation function *d*_2_ (Equation (1)), which encodes the local activity at a given time (*t*), position (*x*, *y*), and correlation time (*τ*). The resulting *d*_2_ images reveal dynamic heterogeneities (**c**), here for the paint droplet at the onset of drying and *τ* = 20 ms.

Laser Speckle Imaging (LSI) was first introduced in the 1980s as a non-invasive and cost-effective imaging technique to visualise subcutaneous and cerebral blood flow^17^,^18^,^19^,^20^. In recent years, its application has been extended to monitoring dynamical heterogeneities in synthetic soft materials^21^,^22^,^23^,^24^,^25^, food technology^26^,^27^, mechanical characterisation of materials^28^,^29^,^30^, and analysis of processes on solid surfaces^31^,^32^,^33^. The technique relies on the illumination of a turbid material of interest with an expanded beam of a coherent light source (Fig. 1a). As photons enter the scattering material, they undergo many scattering events before exiting the sample and reaching a camera. The many scattering events randomise the transport of the photons, resulting in a diffusion path of the photons through the material^34^. The typical randomisation length is given by the transport mean free path l^*^, which can be deduced from Mie theory^35^ or can be determined experimentally^36^. The diffusion coefficient *D*_1_ of light in a multiple scattering medium is *D*_l_ = 3l^*^c/*n*, with *c* = 3 · 10^8^ m/s the speed of light in vacuum, *n* the refractive index of the medium, and l^*^ typically of the order of 10–100 *μ*m, giving 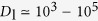 m^2^/s^37^. By contrast, the diffusion rate of scatterers *D*_s_, predicted by the Stokes–Einstein relation, is many orders of magnitude smaller, usually *D*_s_ = 10^−10^–10^−15^ m^2^/s. This large separation in diffusion rates ensures that photons backscattered to the detector essentially probe a static snapshot of the structure. Each photon will travel a different diffusion path through the multiple scattering medium; the resulting path length differences create an interference pattern known as a speckle pattern (Fig. 1b).

As scatterers move within the sample, for instance by Brownian motion, fluid flow, or any other source of structural rearrangements, the speckle pattern changes. In LSI, temporal changes in the speckle intensity are analysed to create imaging contrast. Here we quantify the contrast by the intensity structure function^38^:


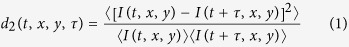


where averaging is over time and/or speckles. The structure function reflects changes in the intensity, with high values indicating decorrelation of the signal and hence enhanced dynamic activity. Note that, when using *d*_2_ as the image contrast, absolute speckle intensity values are irrelevant; even in samples with no contrast in the static structure (Fig. 1b), contrast in local dynamics can emerge (Fig. 1c). We consistently use *d*_2_ to create spatial maps of the dynamic activity rather than the more common autocorrelation function *g*_2_, given by


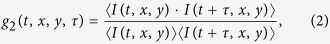


because *d*_2_ was shown both theoretically^38^ and experimentally^39^ to be less sensitive to low-frequency noise and drifts in the intensity. In the case of a stationary ergodic process with sufficient sample size, the two are directly related according to: *d*_2_(*t*, *x*, *y*, *τ*) = 2(*g*_2_(*t*, *x*, *y*, 0) − *g*_2_(*t*, *x*, *y*, *τ*))^38^.

The fact that dynamical heterogeneities in drying paints can be imaged based on temporal speckle correlations was first demonstrated by Zakharov & Scheffold^23^. However, to benefit from the wealth of previously unexplored quantitative information captured in the speckle statistics, we aim to develop Laser Speckle Imaging into a four-dimensional (*t*, *x*, *y*, *τ*) imaging tool that allows a more versatile and extensive analysis of coatings of all types. In particular, we show here that the correlation time *τ* acts as a tuning knob for creating image contrast. This parameter sets the time lag between images that are autocorrelated, and hence the typical time scale of the dynamics of focus. Calculating *d*_2_ for different correlation times allows distinguishing between different types of motion, because the image contrast is highest for dynamics occurring at the *τ* of choice. Additional information about the governing processes can be extracted from the specific shape of the *d*_2_(*τ*) or *g*_2_(*τ*) curves and the associated mean square displacements 〈Δ*r*^2^(*τ*)〉. For example, 〈Δ*r*^2^〉 ∝ *τ*^2^ indicates pure ballistic (deterministic) motion, whereas 〈Δ*r*^2^〉 ∝ *τ* indicates either pure diffusive (stochastic) motion or intermittent local structural changes^40^. Subdiffusive transport is characterised by a power-law exponent smaller than 1. Most practical applications involve a mixture of different types of motion, which manifest as distinct scaling regimes in the mean square displacement (see Supplementary Figs S1 and S2).

To quantify dynamics over a wide range of correlation and experimental times, access to long-term measurements at high frame rates is required. The lower-bound resolution to both *t* and *τ* is determined by the frame rate of the camera; the upper-bound resolution by the total measurement time. We have therefore implemented a real-time distribution feed to stream images of >10^5^ pixels at 100 fps for many hours, yielding seven decades of dynamic range. Note that the lower-bound time resolution is increased when constructing *d*_2_ maps without spatial averaging (e.g. Fig. 1c), as this requires averaging the speckle fluctuations over a finite time interval 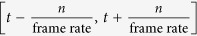 with *n* a positive integer number of frames, effectively equating the time resolution to the width of this interval, 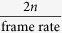.

The spatial resolution is set by either l^*^ or the speckle size on the camera chip divided by the image magnification *M*, depending on which is largest. The minimum speckle size is estimated as *r*_s_ ≈ 1.2*λ*(1 + *M*)*d*/*a*, with *λ* the illumination wavelength, *d* the distance between camera chip and diaphragm, and *a* the aperture diameter^41^,^42^. In our experiments, *r*_s_/*M* = 5–10 *μ*m, implying that in many cases the transport mean free path is limiting to the resolution. Nevertheless, the amount of movement that can be detected, i.e. the displacement resolution, is many orders of magnitude smaller, at the nanometre scale, as multiple scattering amplifies the effects of small displacements^34^.

The effective penetration depth of light into the sample depends on the value of l^*^. For an isotropic sample in the backscattering geometry, most photon paths do not exceed a few l^*^, because the path length (*s*) dependence of the photon flux *J*(*s*) decays as *s*^−5/2^ for large path lengths:


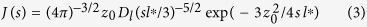


where *z*_0_ ∝ l^*^ is the extrapolation length which contains information about the reflectivity at the sample surface^43^,^44^. The measured correlation functions are thus intensity-weighted convolutions of dynamics over the *z*-axis of the sample, in addition to the averaging in the *xy*-plane dictated by the spatial resolution. l^*^ is in turn a function of the wavelength of the laser. Near-infrared light can achieve penetration depths of millimetres and is most often used for medical imaging, for example to visualise subcutaneous blood perfusion^45^,^46^. By contrast, we use a laser source of 532 nm to achieve an effective penetration depth of the order of tens of micrometres.

### Open time of paint films

We start by exploring a quantitative determination of the open time of paint films. The open time is defined as the time after application beyond which further reworking of the paint film results in visible surface defects. This open time is a crucial handling parameter for all paints, either protective or decorative, but of particular concern for the development of high-quality, sustainable, water-based formulations to replace solvent-based coatings. The eradication of all solvents from a formulation relying strictly on water as the suspending fluid directly causes a strong reduction in the open time, and hence in a loss of application flexibility^47^. Surprisingly, no standardised method exists to determine the open time without ambiguity, despite its importance in the handling properties of a paint. In fact, measurements performed by different operators or techniques often result in dissimilar values. Thus, accurately defining the open time is an important challenge.

To do so with LSI, we apply a homogeneous film of a commercial, pigmented water-based paint on a glass surface using a film applicator to establish the thickness. We then image the centre of the drying paint film, at a controlled temperature of 23 ± 1 °C and relative humidity of 41 ± 2%. As we observe no spatial heterogeneities (Supplementary Fig. S3a) we can average over all speckles in the field of view (>10^5^) to obtain with excellent accuracy the intensity autocorrelation function *g*_2_(*τ*) (Equation (2)) as a function of drying time. Additional time averaging is not necessary, implying that we calculate a true ensemble average. Note that we here use *g*_2_ instead of *d*_2_, because the multi-speckle processing provides ample statistics and moreover, *g*_2_ is more common and therefore offers a more intuitive interpretation.

**Figure 2:**
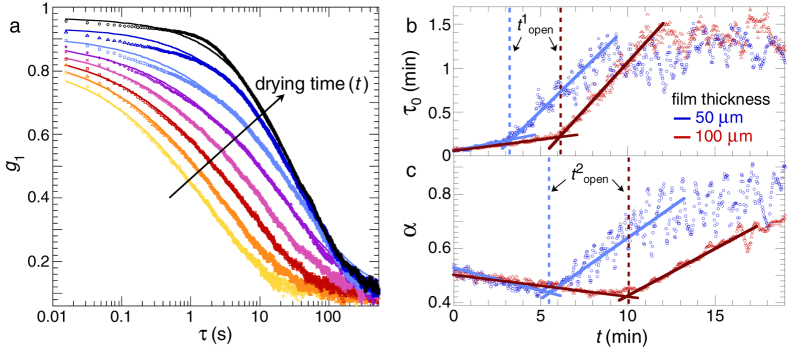
Unambiguous determination of the open time of paint films. (**a**) Evolution of the multi-speckle averaged field autocorrelation function during drying of a 100 *μ*m thick film. For clarity, only a small subset of drying times is shown: *t* = 0 min (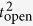), 3.3 min (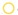), 6 min (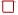), 7.3 min (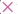), 9.3 min (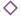), 11.5 min (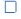), 15 min (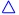) and 20 min (○). The solid lines are fits to a single-exponential decay (Equation (4)), from which the typical time of particle relaxation *τ*_0_(*t*) is obtained. (**b**) Evolution of *τ*_0_ during drying of films of two different thicknesses. The kink in the *τ*_0_ curve marks a transition from a weak to strong rise in viscosity, and hence provides a measure for the open time, 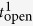. (**c**) Stretching exponent *α* of the fits versus drying time. The minimum in the *α* curve marks a transition from Brownian to ballistic dynamics, indicating that particle diffusion is halted and evolves to advective compaction of a solid-like film. This time thus represents an upper bound to the workability of the film, which we denote by 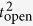.

As time progresses, and more water evaporates from the film (Supplementary Fig. S3b), the correlation function shifts to larger *τ*, indicative of a slowing down of particle diffusion (see Supplementary Fig. S3c,d for *g*_2_(*t*, *τ*) and *d*_2_(*t*, *τ*) curves). We use the Siegert relation to calculate the normalized field autocorrelation functions *g*_1_ from *g*_2_ and fit 600 of these correlation functions to a single-exponential decay, allowing us to extract the time evolution of the characteristic relaxation time *τ*_0_:


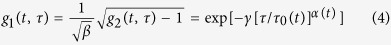


where *α*(*t*) is the stretching exponent, and *β* and *γ* are numerical constants that we have experimentally determined (see ‘Data analysis’)^34^,^48^. Eight typical *g*_1_ curves and fits are shown in Fig. 2a. Each of the 600 fits is based on 4500 data points and has a sum of squared errors SSE below 0.06 (see Supplementary Fig. S3e), enabling an accurate determination of the open time. Note that the characteristic time scale *τ*_0_ is independent of the details of the experimental set-up and requires no pre-existing knowledge of the properties of the sample as the l^*^ dependence is lost in the backscatter geometry. It thus allows a truly unambiguous and reproducible definition of the mobility of particles within the film.

A few minutes after applying the paint, a distinct change in the dynamics appears, which we quantify as a function of film thickness in Fig. 2b. The *τ*_0_ curves display a kink indicative of a transition from a weak to steep increase in film viscosity. Because the workability of a paint film is strongly dependent on viscosity, we can extract a measure for the open time 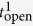 from the position of the kink, as illustrated in Fig. 2b. Remarkably, also the curves of the stretching exponent *α* display two distinct regimes: in the first drying stage, the dynamics are predominantly Brownian, and gradually change from diffusive (*α* = 0.5) to subdiffusive (*α* < 0.5) (Fig. 2c). In the subsequent stage, the particle transport is increasingly governed by advective processes (*α* → 1) arising from the evaporative flux which further concentrates the sample. This crossover from Brownian to advective dynamics signals the transition from a fluid paint film to one with semi-solid properties. Any defect created in a solid-like film can no longer spontaneously anneal; hence, the minimum in the *α* curve marks an upper bound to the workability and consequently a second unambiguous measure for the open time, 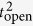.

### Stages in paint drying and aging

**Figure 3:**
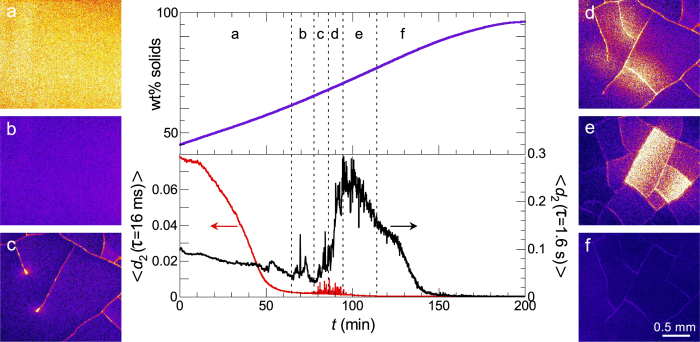
Drying and aging stages of a non-film-forming polymer dispersion on glass, homogeneously coated as a 200 *μ*m thick film. Images (**a**–**f**) show the fast dynamic activity, *d*_2_(*τ* = 16 ms), in the centre of the film in different stages: (**a**) evaporation, (**b**) stress build-up, (**c**) primary cracking, (**d**) secondary cracking, (**e**) delamination, and (**f**) relaxation. All images have the same colour scale and scale bar. See Supplementary Movies S2 and S3 for the time evolution of cracking and delamination. The graphs show that the increase in wt% solids coincides with a shift from fast dynamics, 〈*d*_2_(*τ* = 16 ms)〉 (left ordinate), to slow dynamics, 〈*d*_2_(*τ* = 1.6 s)〉 (right ordinate). Note that the absolute *d*_2_ values inherently increase with increasing *τ*.

In many cases, paint film drying is more complex, exhibiting not only a liquid–solid transition at the open time, but also spatiotemporal heterogeneities and instabilities beyond this point. Identifying these stages is an important step towards unraveling the microscopic mechanisms with which paints dry and become unstable over time. We exemplify this by the drying of a commercial water-based resin dispersion containing polymers that are glassy at room temperature (glass–liquid transition temperature *T*_g_ = 45 °C). LSI images created with *d*_2_ as the contrast function show how the drying dynamics slow down, followed by the appearance of cracks^49^,^50^,^51^ and distinct delamination events in which entire coating fragments detach from the glass substrate^9^ (images a–f in Fig. 3).

By choosing an intermediate value of the correlation time, *τ* = 16 ms, we probe a mixture of diffusive and advective motion. The average value of the contrast function, 〈*d*_2_(*τ* = 16 ms)〉, shows how transport slows down substantially during the first drying stage and arrests fully after approximately 60 minutes (red curve in Fig. 3). At this stage, not yet all water has evaporated, but the suspension reaches a particle concentration of approximately 60 wt% corresponding to random close packing, beyond which particle diffusion and advection are halted. Further water evaporation has no significant effect on the value of *d*_2_ for short *τ*. By contrast, selecting a *τ* two orders of magnitude larger at 1.6 s enables us to create contrast based on slow dynamics within a solid-like paint film, e.g. resulting from capillary deformation of the particles and the nucleation and growth of cracks. While these phenomena do not occur at times <60 minutes, a large burst of these slow aging and cracking dynamics is visible after ~100 minutes. Finally, upon full evaporation of the water, all activity in the film is quenched and a static state is reached.

These data illustrate how *τ* can be used as a spectral parameter to explore different processes and distinguish the successive stages of drying: a) evaporation, b) build-up of shrinkage stresses, c–d) formation of primary and secondary cracks, e) delamination and f) final relaxation of the film by particle deformation. Depending on the stages of interest, the value of *τ* can be fine-tuned to probe specific processes, thus allowing a tailored comparison of different samples or conditions.

### Coffee-ring effect

Additional complexity arises when the geometry of drying is changed from a homogeneous film, in which boundary effects are negligible, to that of a droplet^52^. The drying of dispersion droplets is encountered in a wide variety of scenarios, ranging from spray painting^53^ and inkjet printing^54^ to the drying of blood splatter^55^. As the evaporative flux is higher at the pinned contact line of the droplet, a net flux is created from the centre of the droplet to its periphery. This fluid flow transports solutes to the outer edge, where they are deposited. This so-called coffee-ring effect was explored and explained in detail previously^1^,^56^,^57^,^58^, but only for dilute suspensions to avoid multiple scattering and enable optical imaging. With LSI we can study even the most opaque systems. We therefore image a concentrated droplet of latex particles during drying.

**Figure 4:**
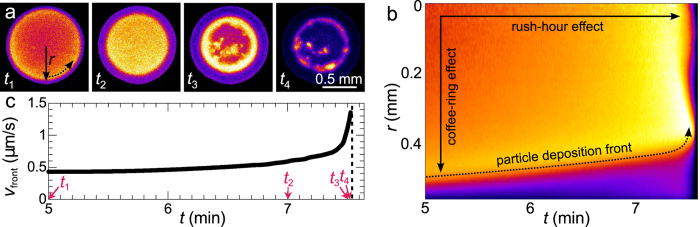
Spatial and temporal heterogeneities in a drying dispersion droplet on glass. (**a**) Time-lapse *d*_2_(*τ* = 10 ms) images of evaporation. All images have the same colour scale and scale bar. See Supplementary Movie S4 for the full time series. (**b**) Kymograph of the azimuthally averaged *d*_2_ versus distance from the centre of the droplet *r* (see (**a**)). The spatial divergence of *d*_2_ by the coffee-ring effect and temporal divergence of *d*_2_ by the rush-hour effect are indicated. (**c**) Velocity of the particle deposition front *v*_front_ = Δ*r*/Δ*t* over time, derived from (**b**). The numbered arrows indicate the evaporation times displayed in (**a**).

In our LSI images we clearly distinguish two key aspects of the coffee-ring effect (Fig. 4a). First, we observe how a dense ring of colloids is deposited at the contact line of the droplet, evident as a depletion of activity at the periphery, which grows inward as time progresses. While the short-time dynamics within this coffee ring are substantially quenched with respect to the liquid centre, where particles diffuse and flow freely, some particle motion is still visible which we attribute to low-frequency particle rearrangements and deformation due to capillary forces. Moreover, we observe a distinct gradient in particle mobility from the centre of the droplet to the deposition front; this is clearly apparent in a plot of the azimuthally averaged value of *d*_2_ as a function of distance to the centre *r* and time *t* (Fig. 4b).

Secondly, the fluid flux to the contact line diverges near the end of drying, which was demonstrated and called the ‘rush-hour effect’ in previous work^57^. The origin of this effect is the increasing area-to-volume ratio of the droplet during drying, which diverges when the last bulk water evaporates and the water contact angle vanishes. We observe a proxy for this effect as a strong rise in the rate with which the coffee ring is deposited. To quantify the propagation velocity of the front, we determine the position of the dense particle deposit from the kymograph in Fig. 4b, defined as the distance *r* where the gradient in *d*_2_ is the largest. Indeed, the front velocity *v*_front_ is virtually constant in the early stages of drying but speeds up strongly as the last bulk water evaporates (Fig. 4c).

Finally, we note that distinct heterogeneities in the particle dynamics emerge in the azimuthal direction mainly towards the end of drying, when the overall particle volume fraction within the droplet approaches the colloidal glass transition (Fig. 4a, *t*_3_ and *t*_4_).

### Paint application

In a drying droplet, spatiotemporal heterogeneities arise as a direct consequence of the droplet geometry. Yet, inhomogeneous dynamics may also result from the method of paint application; in many cases, both consumer and artist paints are applied by means of a paint brush. Since the hairs of a paint brush will not all be identical, this can result in subtle variations in local shear forces, depending on the application pressure, which will affect the drying process.

**Figure 5:**
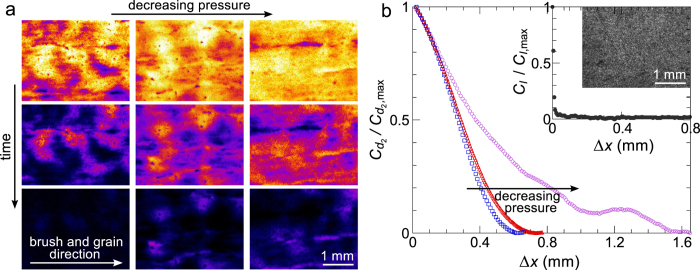
Drying of brush-applied woodtrim paint on plywood. (**a**) Time-lapse *d*_2_(*τ* = 8 ms) images of the centre of paints applied at three different brush pressures. From top to bottom, *t* = 0, 100 and 250 s after application. All images have the same colour scale and scale bar. See Supplementary Movies S5 and S6 for full time series. (**b**) Normalized spatial correlation of *d*_2_ (see text) versus Δ*x* for the measurements in (**a**). The inset shows a white-light photograph of the paint applied at intermediate pressure and the corresponding normalized spatial correlation of the raw intensity versus Δ*x*.

To explore the influence of the application method, we apply a commercial decorative woodtrim paint to a piece of untreated plywood using a hog bristle paint brush. Upon changing the application pressure, no visible changes occur in the appearance of the paint film, implying that variations in the thickness are at the microscopic level. However, when we use LSI to create contrast based on particle dynamics, rich spatial heterogeneities emerge (Fig. 5a). Specifically, the inhomogeneity becomes more pronounced as the brush pressure is increased; this likely originates from an increase in the intra-film thickness variations. To quantify the typical length scale of such dynamical heterogeneities, we calculate the spatial autocorrelation function of *d*_2_, defined as 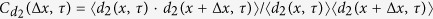. Since brushing is a directional application method, we compute the correlation in the brush direction *x*, while averaging in the perpendicular direction. At short distances, the dynamics are correlated, yet the correlation decays with increasing Δ*x*. From these functions we indeed conclude that the typical domain size grows with decreasing brush pressure. We note that the same analysis performed on the absolute intensity of these samples under white-light illumination reveals no spatially correlated structure (inset Fig. 5b), thus highlighting the purely dynamic nature of these effects.

The dynamical heterogeneities resulting from the initial paint application persist throughout the drying process (Fig. 5a), and also the spatial autocorrelation functions remain remarkably constant. Hence, the manner in which a paint film is applied must be taken into account to truly understand paint drying and its subsequent stability, for which aim LSI is ideally suited.

### Real paints and surfaces

**Figure 6:**
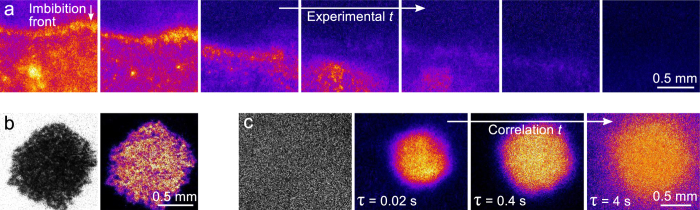
Examples of drying coatings on porous substrates. (**a**) Propagation of an imbibition front through paper. The total time from left to right is 2 minutes. See Supplementary Movie S7 for the full time series. (**b**) Raw speckle image and *d*_2_(*τ* = 10 ms) image of a just-deposited black ink droplet on paper. (**c**) Raw speckle image and *d*_2_(*τ* = 20 ms) images of a white dispersion droplet on white paper, 1 minute after deposition. The *d*_2_ images display the activity for different *τ* values, with the colour scales normalized to the highest *d*_2_ of each image.

To demonstrate the versatility of Laser Speckle Imaging, we explore three practical applications involving highly porous substrates. Interestingly, even if the paint itself is transparent, its dynamics can be inferred if the substrate participates in the drying process. Coatings applied on paper dry not only by evaporation, which is relatively slow, but also by imbibition of the solvent into the porous substrate^59^,^60^. This imbibition will cause the cellulose fibres which constitute the paper to swell and deform^15^, thus creating very small changes in the local structure that can be visualised by LSI. The propagation of such an imbibition front through a piece of paper is shown in Fig. 6a. In sharp contrast to drying on a sealed substrate as in Fig. 4a, the propagation front is rough instead of smooth and its velocity is heterogeneous in time and space. LSI thus allows deducing the fluid dynamics both qualitatively and quantitatively from the substrate mobility.

In addition to transparent systems, we can go to the other extreme and study strongly pigmented systems. The drying dynamics of a just-applied droplet of carbon black ink on paper are revealed in Fig. 6b. Even though the light absorption is strong, provided that sufficient light is multiply scattered we can analyse the drying process. The absolute scattered intensities clearly do not play a role, as 
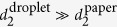
 while *I*^droplet^ ≪ *I*^paper^ (Fig. 6b). Due to the rapid drainage of water from the ink into the paper, the drying is highly heterogeneous and the porosity of the substrate projects in the *d*_2_ image. The imbibition front displays a viscous finger-like pattern characteristic of fluid penetration through a porous material^60^,^61^,^62^.

Finally, we investigate how *τ*-resolved *d*_2_ imaging can provide additional contrast and shed light onto the heterogeneous drying phenomena on paper. Even in scenarios where the raw speckle image is completely devoid of structural information, using LSI we can elucidate how the droplet dries and disentangle different types of processes (Fig. 6c). We achieve this for a single raw speckle movie of a drying paint droplet on paper by calculating *d*_2_ images as a function of correlation time. With increasing *τ*, we shift our focus from fast diffusion and water flows inside the droplet, to intermediately fast processes such as coalescence of particles in the droplet, to very slow dynamics such as swelling of the paper fibres upon imbibition of the water (Fig. 6c). Although the absolute *d*_2_ values inherently increase with increasing *τ*, the spatially resolved *d*_2_ mapping allows distinguishing relative differences in *d*_2_ between different locations in the sample. We note that more thorough analysis of the specific shape of the *d*_2_(*τ*) curves is required to differentiate paint dynamics from substrate dynamics, which is particularly relevant at the droplet periphery where the paint film is so thin that part of the photons will diffuse into the paper. This *τ* dependence thus adds an extra dimension to the three-dimensional raw data, providing a four-dimensional phase space of parameters to choose from, depending on the specific question.

## Discussion

In this paper we have shown how Laser Speckle Imaging is well suited to quantitatively visualise a plethora of complex phenomena that emerge during paint drying and aging. This opens up many new possibilities for the rational and guided design of new sustainable alternatives to solvent-based paints, as well as for unraveling the origins of mechanical instabilities that threaten the longevity of protective coatings and works of art. The modular design of the LSI set-up enables its combination with other techniques; for example, complementing LSI with infrared spectroscopy will allow linking the occurrence of chemical reactions in a paint film, such as those causing artist oil paintings to cure and become brittle over time, to the mechanical stability of the painting. Not only the experimental set-up but also the data processing algorithms can be tailored according to the specific application. We have highlighted the largely unexplored potential of the correlation time *τ* as a spectral tuning knob to obtain image contrast. It is furthermore important to consider the type and extent of averaging: for qualitative imaging, the maximum spatial resolution can be pursued at the expense of temporal resolution, whereas for quantitative mapping, the spatial statistics must be included in the processing to ensure multi-speckle averaging and to suppress speckle noise. High-speed cameras and high speckle densities are desired in this respect – provided that the speckle size exceeds the pixel size – because the statistical noise, expressed by the variance 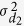 or 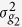, is inversely proportional to the acquired number *N* of independent values of *d*_2_(*τ*) resp. *g*_2_(*τ*)^39^,^63^.

We believe that these initial results hold great promise for more in-depth studies of paints, including their spatially resolved viscoelastic properties, the effects of key parameters on drying and aging, and the physics of fracture. LSI experiments are typically fast, non-destructive and well reproducible under controlled conditions. Moreover, the versatility of Laser Speckle Imaging extends far beyond paints, making it a powerful technique for imaging dynamics of virtually any multiple scattering system.

## Methods

### Experimental set-up

LSI measurements are performed on the home-built set-up shown schematically in Fig. 1a. The illumination source is a 532 nm solid-state laser (Cobolt Samba, 100 mW), whose intensity is regulated by passing the beam through a half-wave plate and polarising beam splitter cube; the latter decomposes the beam into two perpendicular polarisation components, one of which is directed into a beam dump. Adjustment of the rotation angle of the half-wave plate thus allows controlling the intensity of the transmitted component. After reflection by a mirror, the beam is expanded to a diameter of ~1 cm by a Galilean beam expander. The beam is then directed downward onto the sample via two mirrors, at a small angle with respect to the detection path to avoid intensity enhancement by coherent backscattering^36^,^64^. The backscattered light is reflected by a mirror onto a linear polariser perpendicular to the polarisation of the incident laser beam, to filter specular and low-order scattering paths. The multiply scattered light is then collected by a Qioptiq zoom lens and focused through an iris diaphragm and extension tubes onto a CCD camera. To ensure that the speckle statistics are independent of l^*^, the camera detects backscattered photons from the centre of the illuminated area, and all our samples have a thickness ≫l^*^^65^. The magnification of the imaging system is 1.8× and the depth-of-focus is ~0.1 mm. To optimise the spatial resolution whilst retaining a good signal-to-noise ratio, the speckle size is tuned by the diaphragm to be slightly larger than the pixel size, typically 2–3×^21^,^63^. Two cameras are alternately used: a HiSpec 1 camera (Fastec Imaging) for imaging at frame rates up to 1000 fps, and a Dalsa Genie camera (Stemmer Imaging) for continuous streaming at frame rates of ~100 fps. The exposure time is adjusted to cover the full dynamic range of the camera. In parallel to the LSI measurements, a computer-controlled balance (Sartorius, model WZA224-NC) monitors the sample mass with 0.1 mg resolution and 1 Hz measurement frequency. In addition, the temperature and relative humidity are controlled by a home-built climate chamber enclosing the sample, which simultaneously eliminates air convection and stray light.

### Data analysis

In traditional LSI, contrast is expressed by the level of speckle blurring, defined as the ratio of the standard deviation of the intensity to the mean intensity^66^,^67^. This ratio is reduced in areas with fast motion, or fast speckle fluctuations, which become blurred upon imaging with a finite exposure time. Although this analysis is well suited for temporally homogeneous systems, it falls short for systems whose dynamics vary strongly in both time and space. Revealing the latter requires measuring the intensity structure function, *d*_2_ (Equation (1)), or the intensity autocorrelation function, *g*_2_ (Equation (2)). In both equations, we use symmetric normalization, i.e. we normalize the numerators with the product of mean intensities at times *t* and *t* + *τ* instead of the square of the mean intensity, as this reduces artefacts by drift.

To enable true quantitative determination of the open time, it is crucial to have reasonable estimates for the two numerical constants *β* and *γ*. *β* is the spatial coherence factor that accounts for the number of speckles detected. In the ideal case, when each pixel detects fluctuations of only a single speckle, *β* equals 1^48^. As camera-based detection inevitably involves *β* < 1, we choose *β* such that *g*_2_(*τ*) − 1 → *β* for *τ* → 0. *γ* is a numerical prefactor which we have estimated at 1.5^68^ by mapping diffusion coefficients of polystyrene particles in glycerol–water mixtures of different ratios measured by LSI onto those measured by dynamic light scattering (see Supplementary Fig. S1 and text).

## Materials

All components of the LSI set-up were purchased from Thorlabs, unless specified otherwise. All paints are commercial water-based paints, except the sample in Fig. 4 which is a dispersion of poly(styrene-*co*-butyl acrylate) particles (10 wt%, glass–liquid transition temperature *T*_g_ = 36 °C) whose synthesis was described previously^14^. Glass substrates were obtained from ThermoScientific and used without further treatment. Printer paper was purchased from Pioneer (Ultra White office paper). 3 mm thick, untreated pine plywood was used as substrate for the measurements in Fig. 5. These paints were applied with a coarse Storch AquaGel brush. The paints in Fig. 2 were applied with a Quadruple Film Applicator (Erichsen) obtained from BYK–ALTANA.

## Additional Information

**How to cite this article**: van der Kooij, H. M. *et al*. Quantitative imaging of heterogeneous dynamics in drying and aging paints. *Sci. Rep.* **6**, 34383; doi: 10.1038/srep34383 (2016).

## Supplementary Material

Supplementary Information

Supplementary Movie S1

Supplementary Movie S2

Supplementary Movie S3

Supplementary Movie S4

Supplementary Movie S5

Supplementary Movie S6

Supplementary Movie S7
