# Supplementary material for: Quantitative imaging of heterogeneous dynamics in drying and aging paints
Source: Sci Rep. 2016 Sep 29;6:34383. doi: 10.1038/srep34383 (PMC5041151; doi:10.1038/srep34383)
Supplement: Supplementary Information [file srep34383-s1.pdf]

Supplementary Information for:

Quantitative imaging of heterogeneous dynamics in drying and aging paints

Hanne M. van der Kooij,[a,b] Remco Fokkink,[a] Jasper van der Gucht,[a] and Joris Sprakel*[a]

[a]*Physical Chemistry and Soft Matter, Wageningen University, Dreijenplein 6, 6703 HB Wageningen, The Netherlands*
[b]*Dutch Polymer Institute (DPI), P.O. Box 902, 5600 AX Eindhoven, The Netherlands*

E-mail: joris.sprakel@wur.nl

Calibration of prefactor γ

To determine the open time of paint films, we extract the characteristic relaxation time τ_0 by fitting the field autocorrelation functions to a single-exponential decay: $g_1(\tau) = \exp\left[-\gamma(\tau/\tau_0)^{\alpha}\right]$, where γ is a numerical constant that we have calibrated. $\gamma = 1 + \Delta$ is the distance in units of transport mean free path after which the ballistic light impinging onto the sample is converted into diffusive light inside the sample. The value of Δ depends on factors including the polarisation of the incident and detected light, particle size, and refractive index ratio between sample and surroundings. For isotropic scattering by particles dispersed in a medium whose refractive index equals that of the surroundings, the Milne theory predicts that $\Delta = 0.710$. However, most situations involve deviatory values of Δ and consequently of γ.[1]

Calibration of γ for highly concentrated, complex systems such as paints is unfeasible and therefore requires a different approach. To estimate the value of γ in our experiments, we measure the diffusion coefficients of polystyrene particles suspended in water–glycerol mixtures of different ratios, and fit the resulting D values to those measured by dynamic light scattering (DLS). The samples used for LSI contain 1 wt% of polystyrene and are enclosed in a sealed, glass chamber; the samples used for DLS contain 1×10^{-3} wt% of polystyrene and are measured in standard polycarbonate capillaries of 1.9 mm diameter, on an ALV instrument equipped with an ALV-7002 external correlator and a Cobolt Samba 300 mW DPSS laser operating at a wavelength of 532 nm (detection angle = 90°, $T = 23 \pm 1$ °C).

We use two different sizes of the particles, 0.5 and 1 μm, density-matched in 45:55 H_2O–D_2O, and four different glycerol concentrations for the large particles: 0, 25, 30 and 55 wt%. For each sample we measure the multi-speckle averaged autocorrelation function using our LSI set-up and extract the mean square displacement $\langle\Delta r^2(\tau)\rangle$ according to: $g_1(\tau) = \exp\left[-\gamma\, k_0 \sqrt{\langle\Delta r^2(\tau)\rangle}\right]$, where $k_0 = 2\pi n/\lambda$ is the wave vector. The refractive indices n of the different water–glycerol mixtures are obtained from the literature. The resulting mean square displacements versus the correlation time τ are shown in Fig. S1. Their linear scaling confirms the absence of evaporation and sedimentation during the measurements, implying that Brownian motion is the only type of dynamics occurring, hence the diffusion coefficients can be determined by: $\langle\Delta r^2(\tau)\rangle = 6D\tau$. We fit these diffusion coefficients to the 'true' values measured using DLS, considering that $D_{\mathrm{LSI}} \propto 1/\gamma^2$, and find the optimal fit for $\gamma \approx 1.5$.

We note that this value is strictly valid only for samples of polystyrene particles in water–glycerol mixtures and will not apply perfectly to drying paint films. In fact, the strong heterogeneity of paint drying precludes using a single value of γ for the entire drying process. Nevertheless, the actual deviation of γ from 1.5 will be limited and will change only the absolute values of $\langle\Delta r^2(\tau)\rangle$ slightly.

[1] Bicout, D. & Maret, G. Multiple light scattering in Taylor–Couette flow. *Physica A* **210**, 87–112 (1994).

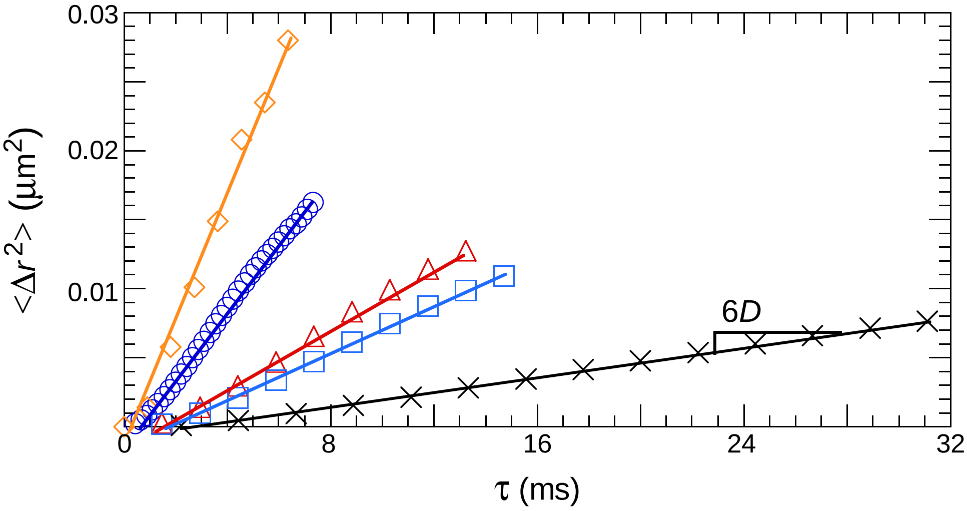

Figure S1: Mean square displacements of polystyrene particles in water–glycerol mixtures with different viscosities, using $\gamma = 1.5$ (see text). The curves correspond to particles of 0.5 μm diameter in pure water (◇); and particles of 1 μm diameter in pure water (○), 25 wt% glycerol (△), 30 wt% glycerol (□), and 55 wt% glycerol (×). The solid lines are least-square fits to the function $\langle \Delta r^2 \rangle = 6D\tau$, from which the diffusion coefficients are obtained.

Differentiation between different types of dynamics

For samples displaying a single type of motion, such as the polystyrene suspensions in sealed sample chambers shown in Fig. S1, where Brownian motion is the only form of dynamics, interpreting the autocorrelation curves and associated mean square displacements is relatively straightforward. Most practical applications, however, involve different types of processes in parallel. An example is the drying droplet in Fig. 4 of the main text. Evaporation of water from the droplet causes not only changes in the diffusion of particles but also a directional flow of particles to the contact line and droplet surface. We here demonstrate the differentiation between these processes in a drying polystyrene suspension droplet using a high-speed camera at 600 fps. The spatially resolved drying dynamics of this droplet are depicted in Fig. S2a. The coffee-ring effect causes the deposition of a dense ring of colloids at the contact line of the droplet, which grows inward as time evolves. A distinct asymmetry appears in the coffee ring after approximately 10 minutes (t_2–t_4). The location where the last bulk water evaporates is delineated by a green circle. To elucidate the governing dynamics over time, we measure the multi-speckle averaged mean square displacements for the circular region (Fig. S2b). The dependence of $\langle \Delta r^2 \rangle$ on τ shows a clear transition from linear (t_1), indicative of diffusive dynamics, to quadratic (t_3–t_4), indicative of ballistic motion. Intermediate times display a mixture of diffusive and ballistic transport (t_2). This shift signifies a decrease in D with increasing concentration and simultaneous increase in advective velocity v due to the coffee-ring effect. We fit the mean square displacements to $\langle \Delta r^2(\tau) \rangle = 6D\tau + (v\tau)^2$, assuming that at t_1 the v term is negligible with respect to the D term, and vice versa at t_3–t_4. This gives a decrease in D from $6 \cdot 10^{-4}$ μm^2/s at t_1 to $4 \cdot 10^{-5}$ μm^2/s at t_2, while simultaneously v increases from 0.12 μm/s at t_2 to 0.21 μm/s at t_3 to 0.32 μm/s at t_4. After evaporation of the bulk water, all fast motion has vanished (t_5) and $\langle \Delta r^2 \rangle$ is negligible in the measured τ range.

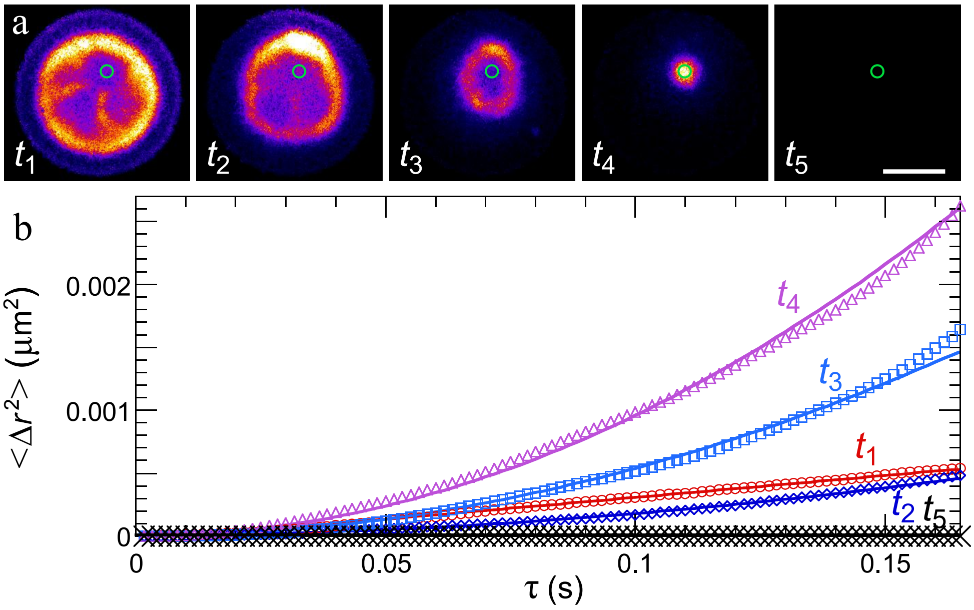

Figure S2: Drying of a polystyrene suspension droplet and quantification of the dynamics inside the green circular region. (a) Time-lapse $d_2(\tau = 1.7$ ms) images of evaporation. t_1 to t_5 represent increasing times after droplet deposition: 7 min (○), 11 min (◇), 13 min (□), 14.3 min (△), and 15.2 min (×). All images have the same scale bar of 0.5 mm and same colour scale except t_1, whose d_2 values were reduced 4×. (b) Mean square displacements obtained by multi-speckle averaging over the 300 speckles inside the green circle, with additional time averaging over 0.4 s to improve the statistics. The labels correspond to the images in (a). The solid lines are least-square fits to the function: $\langle \Delta r^2 \rangle = 6D\tau + (v\tau)^2$.

Open time analysis: evolution of spatial dynamics and film mass, g_2 and d_2 curves, and sum of squared errors of the fits

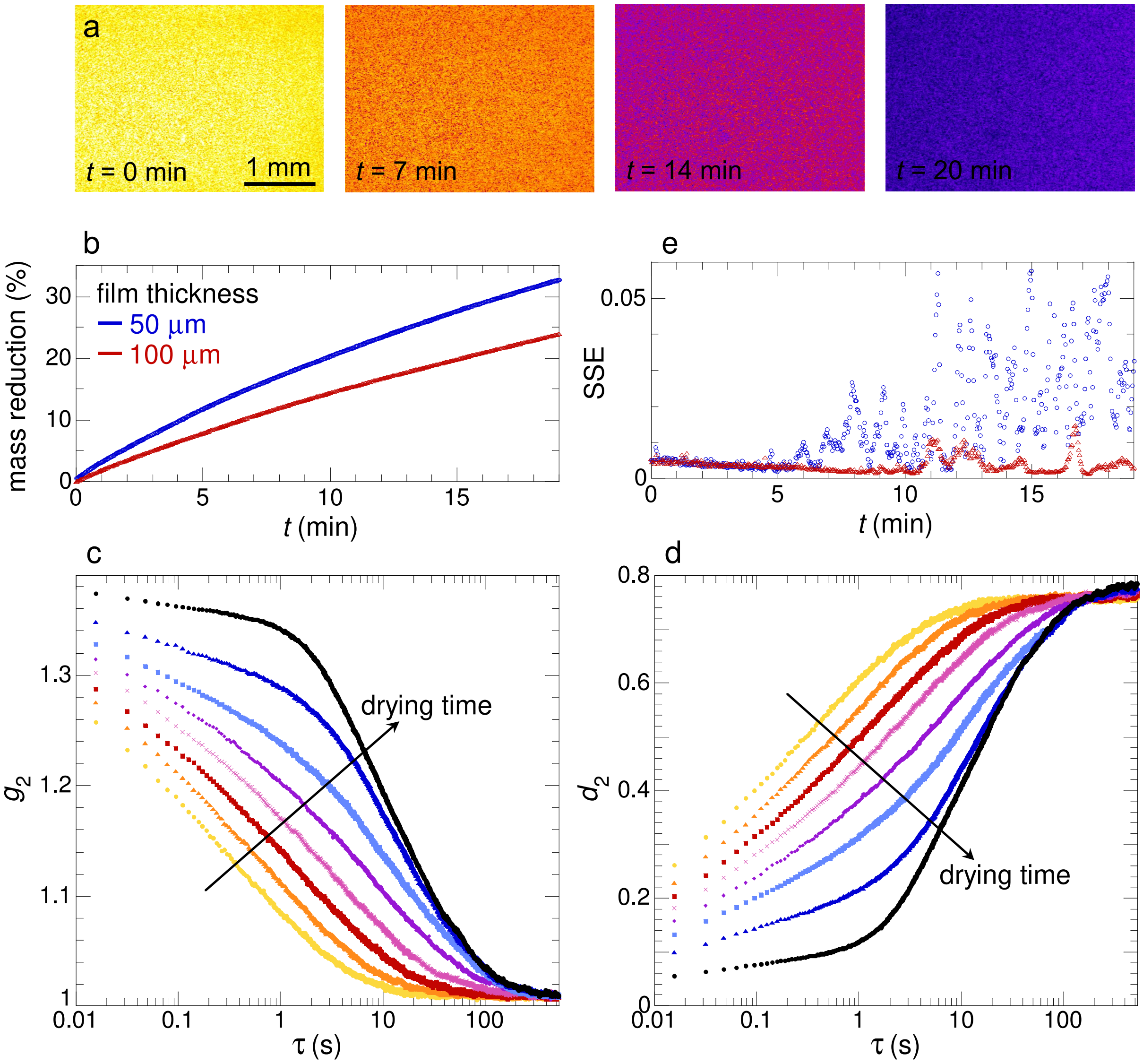

Figure S3: Supplementary data for Fig. 2 of the main text. (a) Spatially resolved $d_2(\tau = 16$ ms) in the centre of a 100 μm thick homogeneous paint film at four drying times. All images have the same colour scale. (b) Reduction of the film mass by water evaporation as a function of drying time for two different film thicknesses. At $t = t^1_{\mathrm{open}}$ and t^2_{open} (compare Fig. 2b–c of the main text), still a considerable amount of water is present in the films. (c) Evolution of the multi-speckle averaged intensity autocorrelation function $g_2(\tau)$ and (d) intensity structure function $d_2(\tau)$ during drying of a 100 μm thick film. The two graphs display the same drying times as shown in Fig. 2a of the main text: $t = 0$ min (●), 3.3 min (▲), 6 min (■), 7.3 min (×), 9.3 min (♦), 11.5 min (■), 15 min (▲) and 20 min (●). All g_2 curves converge towards the intercept $g_2(0) = 1.40 \pm 0.005$, which equals $\beta + 1$. (e) Sum of squared errors SSE corresponding to the single-exponential fits to the g_1 curves, defined as: $\mathrm{SSE} = \sum_{i=1}^{m} [g_1(\tau_i) - f(\tau_i)]^2$ with $m = 4500$ the number of data points per g_1 curve, τ_i the ith correlation time, $g_1(\tau_i)$ the ith g_1 value to be predicted, and $f(\tau_i)$ the predicted value of $g_1(\tau_i)$.

Information about Supplementary Movies

Movie S1) Speckle movie of a drying white paint droplet on white paper
Raw data corresponding to Fig. 1b of the main text. First 4 minutes of drying, 20× real time.

Movie S2) Primary and secondary cracking in the centre of a paint film on glass
Full time series corresponding to Fig. 3c–d of the main text. 5× real time.

Movie S3) Delamination of the centre of a paint film from glass
Full time series corresponding to Fig. 3e of the main text. 5× real time.

Movie S4) Coffee-ring effect in a drying dispersion droplet on glass
Full time series corresponding to Fig. 4a of the main text. t = 5–7.6 min after droplet deposition, real time.

Movie S5) Drying of a paint film on plywood, applied by brush at high pressure
Full time series corresponding to Fig. 5a, left column, of the main text. First 6 minutes of drying, 20× real time.

Movie S6) Drying of a paint film on plywood, applied by brush at low pressure
Full time series corresponding to Fig. 5a, right column, of the main text. First 7 minutes of drying, 20× real time.

Movie S7) Propagation of an imbibition front through paper
Full time series corresponding to Fig. 6a of the main text. 5× real time.
